# Supplementary material for: Using cellular fitness to map the structure and function of a major facilitator superfamily effluxer
Source: Mol Syst Biol. 2017 Dec 1;13(12):964. doi: 10.15252/msb.20177635 (PMC5740499; doi:10.15252/msb.20177635)
Supplement: Supplementary file 1 — Appendix [file MSB-13-964-s001.pdf]

Appendix

Using cellular fitness to map the structure and function of a Major Facilitator Superfamily effluxer

Anisha M. Perez[1,†], Marcella M. Gomez[2,†], Prashant Kalvapalle[3], Erin O'Brien-Gilbert[1], Matthew R. Bennett[1,4,5], and Yousif Shamoo[1,4,*]

[1] Department of BioSciences at Rice, Rice University, Houston, TX 77005, USA
[2] Department of Applied Mathematics & Statistics, University of California, Santa Cruz, Santa Cruz, CA 95064, USA
[3] Systems, Synthetic, and Physical Biology Graduate Program, Rice University, Houston, TX 77005, USA
[4] Institute of Bioscience and Bioengineering, Rice University, Houston, TX 77005, USA
[5] Department of Bioengineering, Rice University, Houston, TX 77005, USA
[†] These authors contributed equally to this work
[*] Corresponding author: shamoo@rice.edu

Table of Contents

Figure S1. Pump parameters κ and γ are sensitive to different parts of the fitness function curve….2

Figure S2. TetB physicochemical-fitness model can tightly fit fitness functions for all variants across TET, DOX and MCN…………………………………………………………………………....3

Figure S3. Insertion and expression of *tet(B)^{WT}* and variants results in modest fitness decrease compared to BW25113 without *tet(B)*…………………………………………………………4

Figure S4. Relative percent change in binding rate, k_1, and pump efficiency rate, *r*, for DOX and MCN relative to TET………………………………………………………………………...5

Figure S5. Triplicate growth curves for BW25113 expressing *tet(B)* variants used to harvest culture samples at mid-exponential growth phase………………………………………………………6

Figure S6. *In vivo* protein levels of TetB variants relative to wild-type were determined by Western Blot analysis of cell membrane fractions…………………………………………………...7

Figure S7. Statistics of ratio γ/κ for all TetB chromosomal variants allow for testing of first order approximation assumptions…………………………………………………………………8

Figure S8. Relative changes in k_1 and *r* for all TetB chromosomal variants uncoupled from κ and γ using *in vivo* protein levels……………………………………………………………………...9

Figure S9. Strains expressing *tet(B)^{V339C}* and *tet(B)^{G366C}* provide minimal fitness increase in MCN...10

Figure S10. Triplicate fitness functions and model fits of *E. coli* strains expressing seven *tet(B)* variants isolated from a *tet(B)* error-prone PCR library…………………………………………...11

Figure S11. Derivation of *E. coli* BW25113 expressing chromosomal *tet(B)^{WT}* fitness function in MCN from logarithmically transformed growth curves……………………………………...…….12

Table S1. Lumped physicochemical parameters of TetB chromosomal variants determined from modeling fitness functions in TET, DOX and MCN…………………………………………….13

Table S2. Lumped physicochemical parameters of TetB chromosomal variants with uncertainty in measured global parameters A and B……………………………………………...…………….…….14

Table S3. Lumped physicochemical parameters of TetB plasmid variants determined from modeling fitness functions in TET…………………..……………………………………….…………………...15

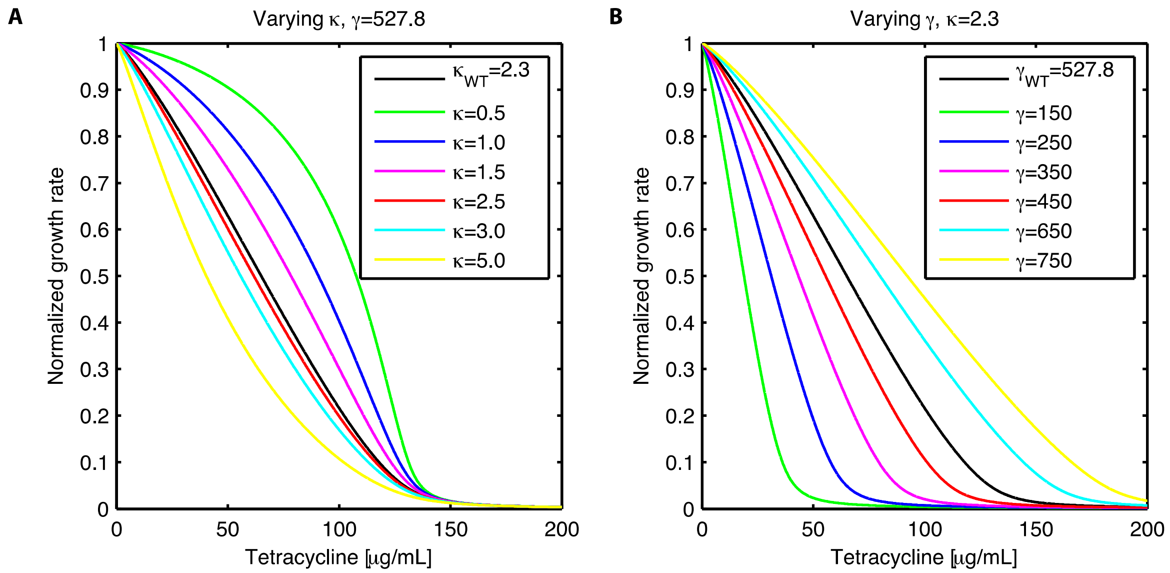

Figure S1. Pump parameters *κ* and *γ* are sensitive to different parts of the fitness function curve. Our physicochemical-fitness model uses fitness functions generated from growth at selective conditions to predict parameters *κ* and *γ*, which roughly describe substrate affinity and total activity, respectively. **A.** The predicted value of *γ* for $TetB^{WT}$, 527.8, is held constant and *κ* is varied. We find that *κ* is sensitive to the initial plateau seen in the fitness function. As *κ*, the apparent K_m, decreases the tighter the binding between substrate and TetB. This tighter binding results in an extension of the initial plateau of the fitness function. **B.** The predicted value for *κ* for $TetB^{WT}$, 2.3, is held constant and *γ* is varied. We find that *γ* is sensitive to the drop off point of the fitness function resulting from the overall ability of TetB to efflux substrate out of the cytoplasm and is influenced by both the total amount of TetB and pumping efficiency rate. The units for both *κ* and *γ* are µg/mL.

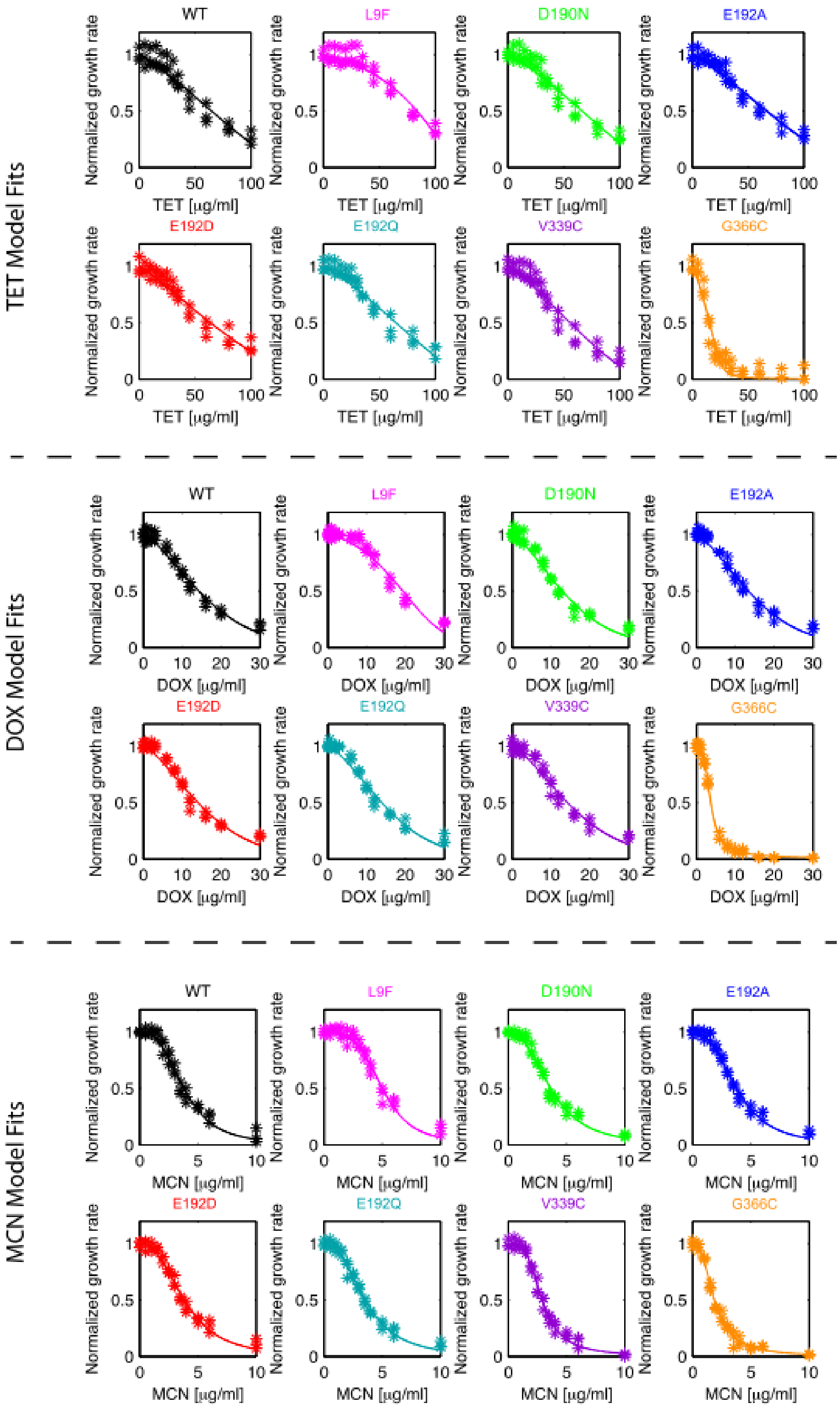

Figure S2. TetB physicochemical-fitness model can tightly fit fitness functions for all variants across TET, DOX and MCN. Shown are triplicate normalized fitness functions (*) and their corresponding model fits (solid line) of strains expressing *tet(B)*^{WT} or chromosomal variant across a range of TET, DOX and MCN concentrations.

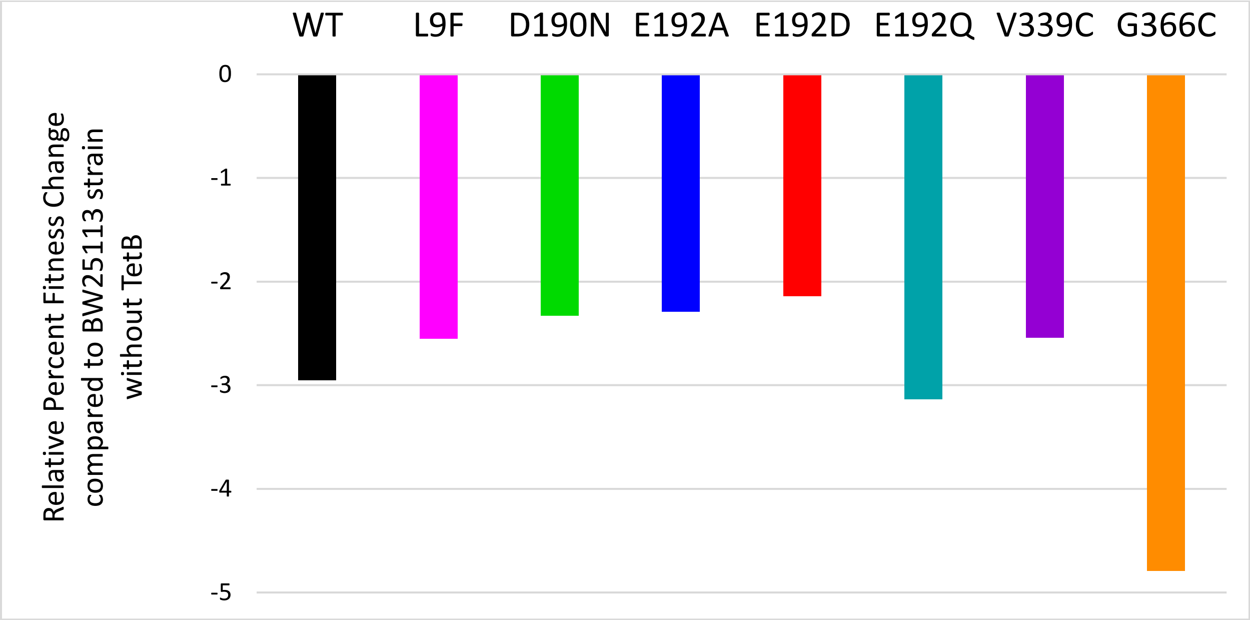

Figure S3. Insertion and expression of *tet(B)*^{WT} and variants results in modest fitness decrease compared to BW25113 without *tet(B)*. The average of the raw, non-normalized growth rates at zero drug concentration were used to determine the percent fitness cost associated with the expression of *tet(B)* compared to host strain BW25113 without *tet(B)*. For the *tet(B)* constructs, the average growth rates were calculated from 9 single colony growth rates at zero drug comprised of triplicate growth rates from each of the TET, DOX and MCN growth assay plates. Similarly, the average growth rates for BW25113 was calculated from 12 single colony growth rates at zero drug comprised of quadruplicate growth rates from each of the TET, DOX and MCN growth assay plates. The following equation was used to calculate the percent fitness decrease:

$$Percent\ Fitness\ Cost = 1 - \left(\frac{GR^{TetB}}{GR^{BW25113}}\right) * 100$$

Percent fitness cost was determined for strains expressing *tet(B)*^{WT} and variants. Most variants displayed a fitness cost between 2% - 3%. The strain expressing *tet(B)*^{G366C} displays the largest fitness cost at ~ 4.8%.

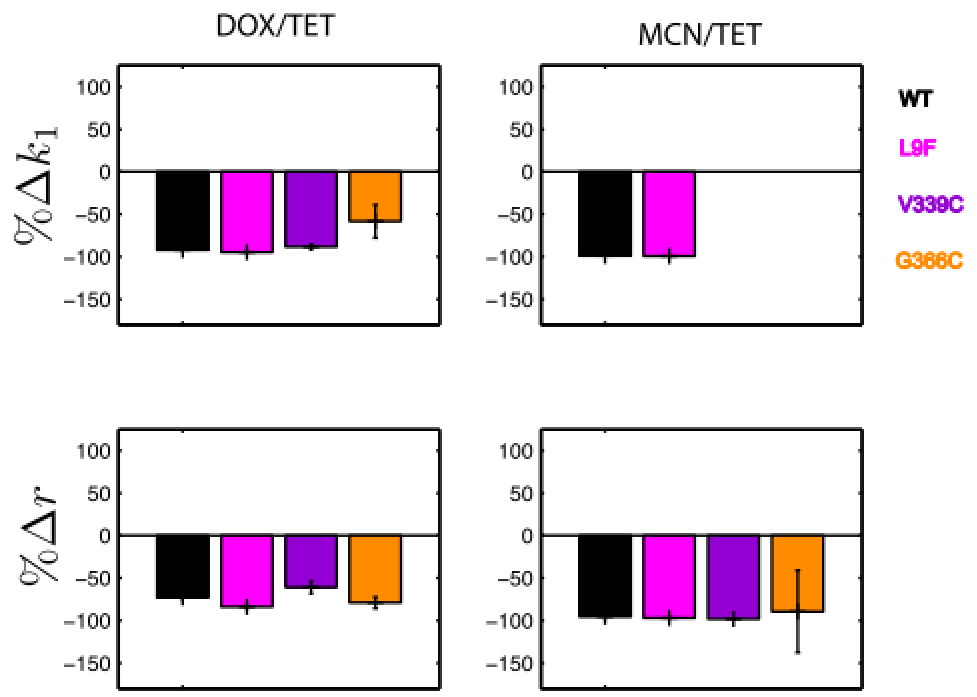

Figure S4. Relative percent change in binding rate, k_1, and pump efficiency rate, *r*, for DOX and MCN relative to TET. The effects of mutations are compared across the different drugs. For wild-type (black) and each mutant $TetB^{L9F}$ (pink), $TetB^{V336C}$ (purple) and $TetB^{G366C}$ (orange), the substrate binding affinity and pumping efficiency with DOX and MCN is compared to performance with TET. This analysis shows that the reduction in binding affinity and pumping efficiency when working against DOX and MCN is comparable across all mutations, although slightly worse for MCN and more variable for DOX. This analysis assumes that diffusion and protein concentration remain constant across the different tetracycline antibiotics. Error bars come from 1000 randomly selected parameters *A* and *B* in parameter fits of κ and γ. Changes in k_1 could not be resolved for MCN for $TetB^{V336C}$ and $TetB^{G366C}$ (See Fig. S7).

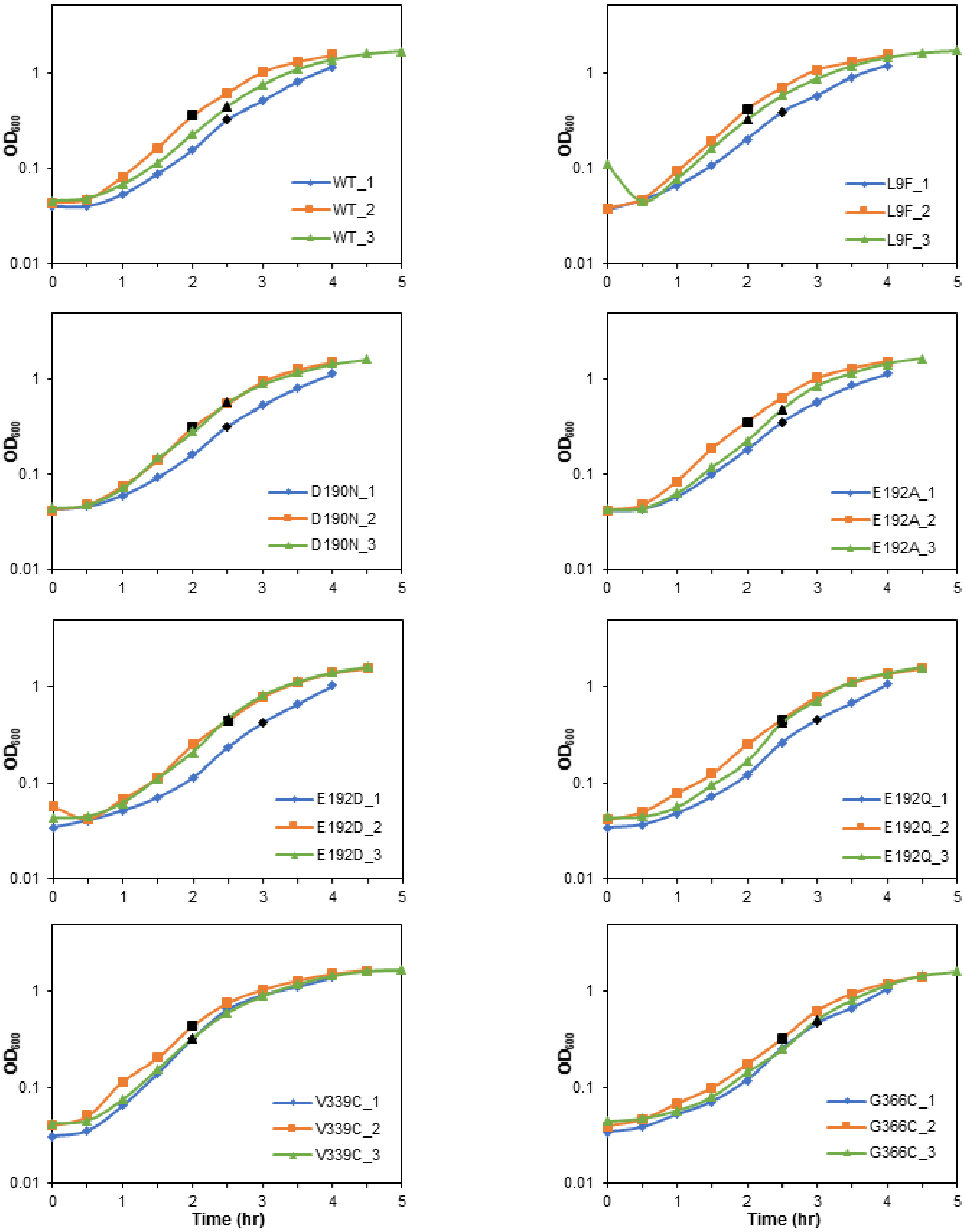

Figure S5. Triplicate growth curves for BW25113 expressing *tet(B)* variants used to harvest culture samples at mid-exponential growth phase. Glycerol stocks of BW25113 were first streaked onto LB agar plates containing 0.18% glucose. Single colonies were then grown for 24hours in 2% arabinose supplemented LB to pretreat the cells and induce *tet(B)* expression. The culture was then diluted 1:100 in fresh 2% arabinose supplemented LB where growth was monitored by measuring the optical density at 600nm every 30 minutes for 4 – 5 hours. Culture sample was collected once cells hit mid-exponential growth phase as indicated by the black marker and was used to determine relative *in vivo* protein levels. Most strains reached mid-exponential growth phase after 2 – 3 hours of growth.

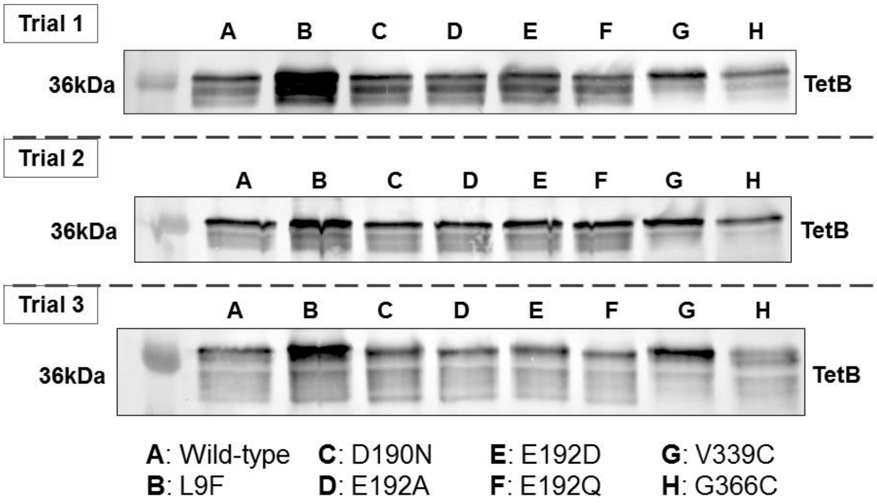

Figure S6. *In vivo* protein levels of TetB variants relative to wild-type were determined by Western Blot analysis of cell membrane fractions. Strains were grown in 2% arabinose in the absence of drug and harvested at mid-exponential phase. Membrane samples were prepared from three individual colonies grown and prepared independently. 45µg of each membrane sample was loaded onto a 10% SDS PAGE gel, transferred onto PVDF membrane and probed with an antibody for TetB. Despite the use of a protease inhibitor cocktail within the lysis buffer used to prepare the samples, degradation of TetB can be seen in all variants. Since it cannot be determined when the degradation occurred, *in vivo* or during preparation, all bands were used to quantitate *in vivo* TetB levels using ImageJ software.

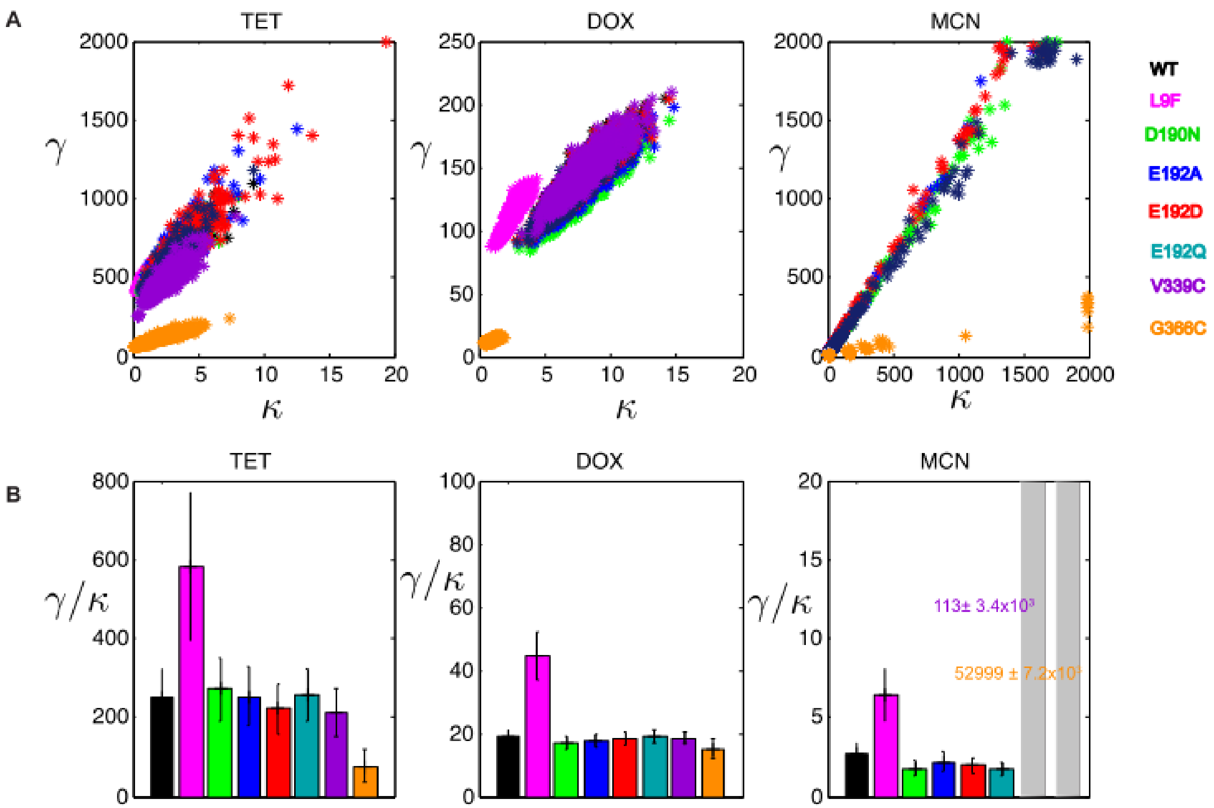

Figure S7. **Statistics of ratio γ/κ for all TetB chromosomal variants allow for testing of first order approximation assumptions. A.** The spread of κ and γ fits for 1000 randomly selected parameters *A* and *B* from a set gamma distribution shows a linear correlation between κ and γ. All interdomain loop mutants cluster and overlap with the TetB^{WT} fits. **B.** The calculated mean and error of γ/κ for the sampled parameter fittings shown in Figure S2. Although an extensive spread of values are seen for the interdomain loop mutants in part A, the strong linear correlation between κ and γ allows us to confidently calculate γ/κ. Changes in γ/κ could not be resolved for TetB^{V336C} and TetB^{G366C} in MCN. This suggests that a linear relationship does not exist and the zero order approximation of Equation (24) does not hold, hence, the reverse binding reaction rate (k_{-1}) is comparable to the pumping efficiency.

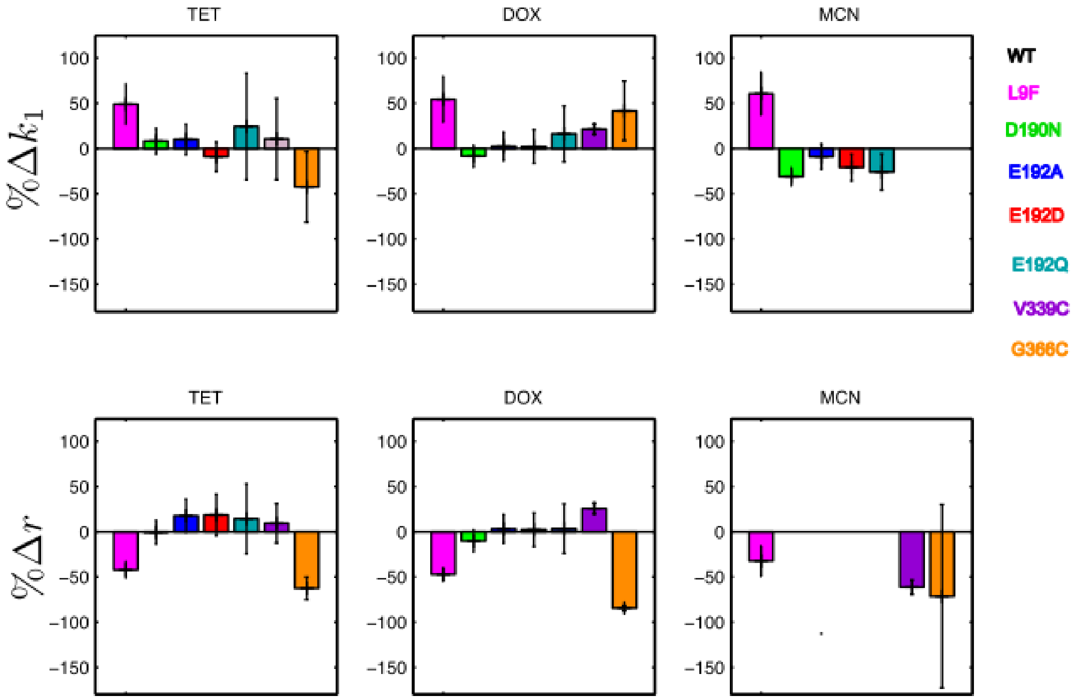

Figure S8. Relative changes in k_1 and r for all TetB chromosomal variants uncoupled from κ and γ using *in vivo* protein levels. Changes in drug binding affinity Δk_1 and pumping efficiency Δr for TetB variants relative to wild type for TET, DOX and MCN. Error bars are comprised from a propagation of errors from *A* and *B* global parameter modeling and *in vivo* protein level determination. The linear correlation between κ and γ allow for the confident determination of Δk_1 for all variants except V339C and G366C. As expected, the interdomain loop mutants which display similar cellular fitness and *in vivo* protein levels to wild-type do not alter drug binding affinity. Changes in pump efficiency is dependent on the confidence in γ parameter and P_{tot} estimation. Δr for the interdomain loop mutants could not be resolved for MCN due to the high variability of the γ parameter.

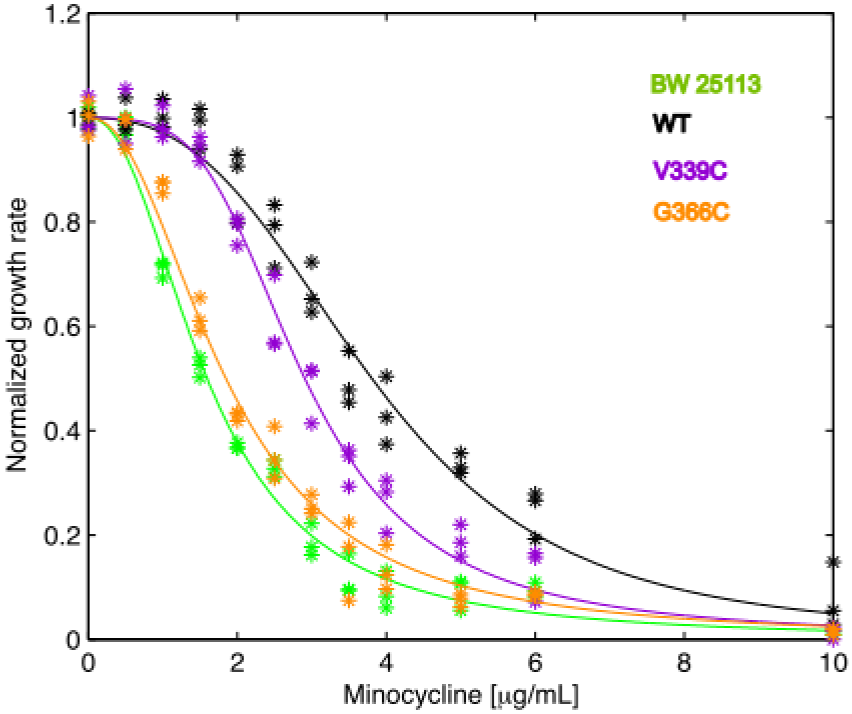

Figure S9. Strains expressing *tet(B)*^{V339C} and *tet(B)*^{G366C} provide minimal fitness increase in MCN. Triplicate fitness functions (*) and model fits (solid line) of host cell BW25113, strains expressing *tet(B)*^{WT}, *tet(B)*^{V339C} and *tet(B)*^{G366C} in MCN. The strain expressing *tet(B)*^{G366C} displays nearly overlapping fitness function to the strain expressing *tet(B)*^{WT}. TetB^{V339C} provides a greater but still minimal fitness increase compared to TetB^{WT}. This minimal fitness increase in hypothesized to be the cause of large error seen in the predictions of kappa for TetB^{V339C} and TetB^{G366C} (Appendix Table S1). This large error is further propagated in the estimation of initial binding rate, k_1, leading to the inability to estimate Δk_1 relative to WT for these two variants (Figure 6).

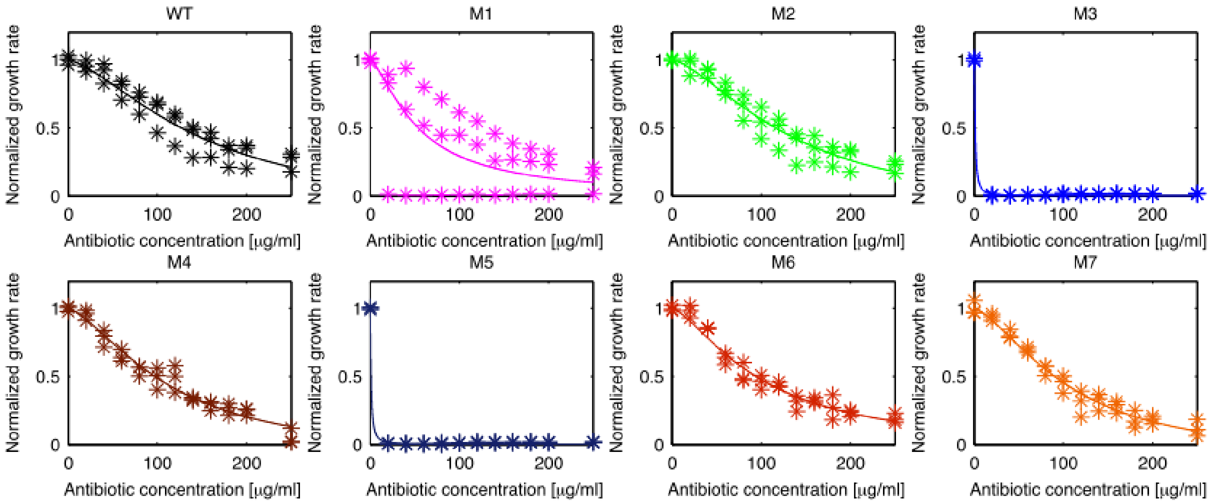

Figure S10. Triplicate TET fitness functions and model fits of *E. coli* strains expressing seven *tet(B)* variants isolated from a *tet(B)* error-prone PCR library. Fitness functions for *E. coli* BW25113 strains harboring pSC101 plasmid copies of *tet(B)*^{WT} and seven plasmid variants in TET. Variants 3 (blue) and 5 (navy) did not grow in the any TET concentrations used in this assay. Variants 2 (green) and 4 (brown) exhibited similar fitness to wild-type while Variant 1 (pink) displayed highly inconsistent fitness. Variant 6 (red) and Variant 7 (orange) have reduced fitness compared to wild-type. Variant 6 harbors three non-synonymous mutations; K183E, A290V, and T326I. Variant 7 also harbors three non-synonymous mutations; F151I, F179L, and T326A.Triplicate growth rate data are plotted in asterisks (*) and the model fits are shown as solid lines.

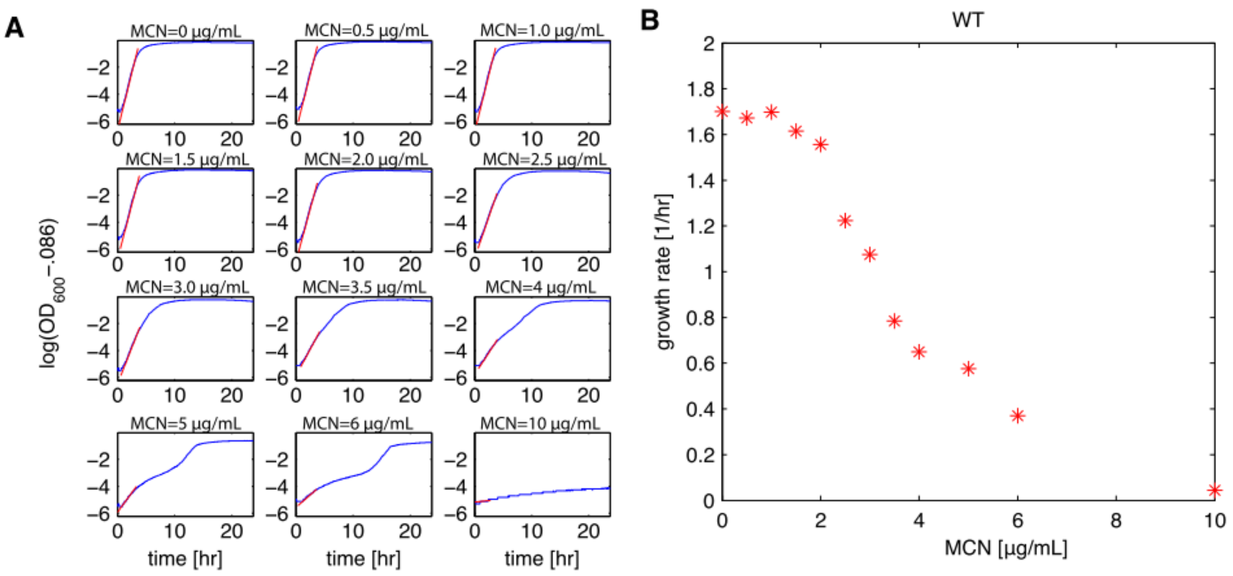

Figure S11. Derivation of *E. coli* BW25113 expressing chromosomal *tet(B)*^{WT} fitness function in MCN from logarithmically transformed growth curves. A. Logarithmic growth curves representing one row of OD_{600} measurements at 12 MCN concentrations within a 96-well plate of BW25113 expressing chromosomal *tet(B)*^{WT}. The growth curves are background corrected for the blank media OD_{600} measurement of 0.086. The slope of the red line tangent to the curve at the first exponential growth phase was taken as the growth rate (*Materials and Methods- Mathematical Analysis*) **B.** Absolute (non-normalized) fitness function of BW25113 expressing chromosomal *tet(B)*^{WT} generated by plotting first exponential growth rate as a function of MCN concentration.

		WT	L9F	D190N	E192A	E192D	E192Q	V339C	G366C
**TET**	κ	2.26	0.89	2.08	2.42	2.92	2.14	2.37	1.76
	γ	527.75	470.73	519.30	562.51	595.67	512.44	462.97	108.61
	error	0.23	0.17	0.20	0.12	0.16	0.17	0.23	0.27
**DOX**	κ	7.65	2.76	7.54	7.74	7.69	6.84	7.94	0.83
	γ	141.50	117.10	125.81	132.85	139.59	128.29	144.63	12.20
	error	0.09	0.11	0.11	0.08	0.12	0.10	0.11	0.02
**MCN**	κ	7.91	3.33	19.21	12.31	17.40	18.43	1.25	0.0001
	γ	19.60	20.69	32.27	25.29	32.77	29.61	6.20	1.02
	error	0.10	0.10	0.07	0.06	0.07	0.06	0.06	0.04

Table S1. Lumped physicochemical parameters of TetB chromosomal variants determined from modeling fitness functions in TET, DOX and MCN. Parameters κ and γ were determined using the average A and B global parameters from Table 1. The error is calculated by taking the square root of the sum of the residual errors squared. This is the objective function used for the parameter fitting. This is the error corresponding to the fits in **Figure S2**.

	TET		DOX		MCN	
	κ	γ	κ	γ	κ	γ
**WT**	2.35 ± 0.93	538.84 ± 82.64	7.72 ± 1.48	142.85 ± 16.00	10.81 ± 21.62	23.46 ± 27.55
**L9F**	0.90 ± 0.31	472.93 ± 32.76	2.67 ± 0.59	115.58 ± 10.87	3.63 ± 1.50	21.35 ± 3.40
**D190N**	2.15 ± 0.84	528.92 ± 74.87	7.61 ± 1.63	127.07 ± 16.42	79.55 ± 234.77	105.77 ± 283.29
**E192A**	2.55 ± 1.14	579.47 ± 110.46	7.81 ± 1.62	134.17 ± 16.67	33.92 ± 130.38	53.80 ± 171.29
**E192D**	3.13 ± 1.55	620.82 ± 148.00	7.74 ± 1.58	140.76 ± 17.12	65.77 ± 203.17	97.59 ± 270.98
**E192Q**	2.26 ± 0.89	528.76 ± 81.51	6.89 ± 1.36	129.36 ± 14.55	92.43 ± 284.18	115.05 ± 325.74
**V339C**	2.43 ± 0.86	469.83 ± 66.05	8.01 ± 1.54	146.02 ± 16.66	1.61 ± 1.34	6.52 ± 1.28
**G366C**	1.85 ± 1.03	111.59 ± 27.67	0.87 ± 0.25	12.42 ± 1.11	34.81 ± 189.80	6.68 ± 30.72

Table S2. Lumped physicochemical parameters of TetB chromosomal variants with uncertainty in measured global parameters *A* and *B*. Parameters κ and γ were determined using a sampling of *A* and *B* global parameters from Table 1. Unsurprisingly, the four variants tested at residues 190 and 192 that had similar fitness functions to WT also have lumped parameter values similar to WT in TET and DOX but have variable lumped parameters with high error in MCN. The errors within the κ parameter for the interdomain loop mutants $TetB^{D190N}$, $TetB^{E192[A,D,Q]}$, $TetB^{V339C}$, and $TetB^{G366C}$ in MCN are very large and therefore we do not have confidence in this estimation. Additionally, the γ parameter for the interdomain loop mutants, also cannot accurately be estimated in MCN. In TET and DOX, we observe that small changes in parameters κ and γ have the ability to produce large fitness effects in our selection conditions. The units for both κ and γ are µg/mL.

TET		TetB Plasmid Variants							
		**1**	**2**	**3**	**4**	**5**	**6**	**7**	**WT**
	κ	443.34	8.05	712.84	9.30	716	23.63	8.05	8.25
	γ	49898	2418.38	991.43	2275.44	991.37	5024.65	1876.42	2697.02
	error	2.40	0.20	0.01	0.12	0.005	0.10	0.09	0.22

Table S3. Lumped physicochemical parameters of TetB plasmid variants determined from modeling fitness functions in TET. Parameters κ and γ were determined using the average A and B global parameters listed in the main text (A=1.91 and B=1.42). The error is calculated by taking the square root of the sum of the residual errors squared. This is the objective function used for the parameter fitting. This is the error corresponding to the fits in **Figure S10**.
