# Supplementary material for: Using cellular fitness to map the structure and function of a major facilitator superfamily effluxer
Source: Mol Syst Biol. 2017 Dec 1;13(12):964. doi: 10.15252/msb.20177635 (PMC5740499; doi:10.15252/msb.20177635)
Supplement: Supplementary file 6 — Source Data for Appendix [file MSB-13-964-s010.zip › SourceData_Appendix/README.rtf]

Source Data for Appendix Figure S10Contents:Two Excel files. One containing Source Data for Figure S10 (plasmid variants) consisting of OD600 readings every 5 minutes for 24 hours.Prior to analysis, a background OD600 of 0.086 must be subtracted from the source data to account for the OD600 of the LB media. The second Excel file contains Analyzed Data for Figure S10 which was analyzed using the Growth rate code included in this manuscipt as Computer Code EV1.
