# Supplementary material for: Using cellular fitness to map the structure and function of a major facilitator superfamily effluxer
Source: Mol Syst Biol. 2017 Dec 1;13(12):964. doi: 10.15252/msb.20177635 (PMC5740499; doi:10.15252/msb.20177635)
Supplement: Supplementary file 8 — Source Data for Figure 1B [file MSB-13-964-s006.zip › SourceData_Figure1B/README.txt]

Source Data for Figure 1: Contents: Two Excel files. One containing Source Data for Figure 1 (BW25113 without TetB) consisting of OD600 readings every 5 minutes for 24 hours.Prior to analysis, a background OD600 of 0.086 must be subtracted from the source data to account for the OD600 of the LB media. The second Excel file contains Analyzed Data for Figure 1 which was analyzed using the Growth rate code included in this manuscipt as Computer Code EV1.This data was also used to obtain global parameters A and B for which Computer Code EV2 was used. 
