# Supplementary material for: Using cellular fitness to map the structure and function of a major facilitator superfamily effluxer
Source: Mol Syst Biol. 2017 Dec 1;13(12):964. doi: 10.15252/msb.20177635 (PMC5740499; doi:10.15252/msb.20177635)
Supplement: Supplementary file 9 — Source Data for Figure 2 [file MSB-13-964-s007.zip › SourceData_Figure2/README.txt]

Source Data for Figure 2Contents: Two Excel files. One containing Source Data for Figure 2 (TetB wild-type) consisting of OD600 readings every 5 minutes for 24 hours.Prior to analysis, a background OD600 of 0.086 must be subtracted from the source data to account for the OD600 of the LB media. The second Excel file contains Analyzed Data for Figure 2 which was analyzed using the Growth rate code included in this manuscipt as Computer Code EV1. This data was also used to obtain lumped parameters kappa and gamma for TetB wild-type using Computer Code EV2.
