# Supplementary material for: Using cellular fitness to map the structure and function of a major facilitator superfamily effluxer
Source: Mol Syst Biol. 2017 Dec 1;13(12):964. doi: 10.15252/msb.20177635 (PMC5740499; doi:10.15252/msb.20177635)
Supplement: Supplementary file 11 — Source Data for Figure 4 [file MSB-13-964-s009.zip › SourceData_Figure4/README.txt]

Source Data for Figure 4Contents: One Excel file which contains the ImageJ analysis of TetB western blot in Figure S6. This analysis was then used to determine TetB chromosomal variant protein levels relative to wild-type shown in Figure 4. 
